# Two Complementarity Immunotherapeutics in Non-Small-Cell Lung Cancer Patients—Mechanism of Action and Future Concepts

**DOI:** 10.3390/cancers13112836

**Published:** 2021-06-07

**Authors:** Kamila Wojas-Krawczyk, Paweł Krawczyk, Michał Gil, Maciej Strzemski

**Affiliations:** 1 Pneumonology, Oncology and Allergology Department, Medical University of Lublin, 20-954 Lublin, Poland; kamilawojas@wp.pl (K.W.-K.); krapa@poczta.onet.pl (P.K.); 2 Genetics and Immunology Institute, GENIM Ltd., 20-609 Lublin, Poland; 3 Analytical Chemistry Department, Medical University of Lublin, 20-093 Lublin, Poland; maciej.strzemski@poczta.onet.pl

**Keywords:** immunotherapy, non-small-cell lung cancer, immune checkpoints, tumour microenvironment

## Abstract

**Simple Summary:**

Here, we focused on the most important mechanisms of action of combined immunotherapy with modern anticancer approaches in patients with non-small-cell lung cancer. This knowledge is extremely important for lung cancer clinicians. First, it facilitates proper involvement of the patient in the treatment and monitoring its effectiveness. More importantly, the knowledge of the immunotherapy mechanisms will certainly allow quick recognition of the side effects of such a therapy, which are totally different of those observed after chemotherapy. Side effects of combination therapies can occur at any stage of treatment, and even after completion thereof. This review article could particularly explain the mechanism of action of combined immunotherapy, which have different targets in patients.

**Abstract:**

Due to the limited effectiveness of immunotherapy used as first-line monotherapy in patients with non-small-cell lung cancer (NSCLC), the concepts of combining classical immunotherapy based on immune checkpoint antibodies with other treatment methods have been developed. Pembrolizumab and atezolizumab were registered in combination with chemotherapy for the treatment of metastatic NSCLC, while durvalumab found its application in consolidation therapy after successful chemoradiotherapy in patients with locally advanced NSCLC. Exceptionally attractive, due to their relatively low toxicity and high effectiveness, are treatment approaches in which a combination of two different immunotherapy methods is applied. This method is based on observations from clinical trials in which nivolumab and ipilimumab were used as first-line therapy for advanced NSCLC. It turned out that the dual blockade of immune checkpoints activated T lymphocytes in different compartments of the immune response, at the same time affecting the downregulation of immune suppressor cells (regulatory T cells). These experiments not only resulted in the registration of combination therapy with nivolumab and ipilimumab, but also initiated other clinical trials using immune checkpoint inhibitors (ICIs) in combination with other ICIs or activators of costimulatory molecules found on immune cells. There are also studies in which ICIs are associated with molecules that modify the tumour environment. This paper describes the mechanism of the synergistic effect of a combination of different immunotherapy methods in NSCLC patients.

## 1. Introduction

**Figure 1:**
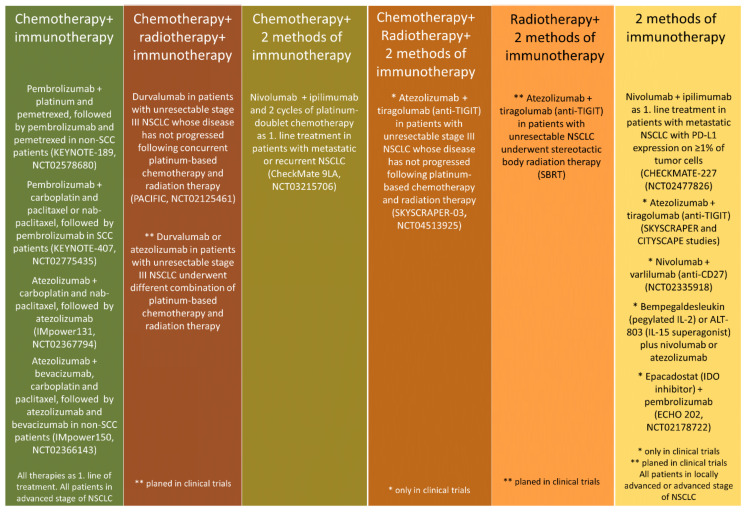
Many new options for combining cancer therapies are already available in NSCLC clinic, others as part of ongoing clinical trials.

Checkpoint inhibitors such as anti-PD-1 (programmed death 1), anti-PD-L1 (programmed death ligand 1), or anti-CTLA-4 (cytotoxic T lymphocyte antigen 4) antibodies are widely used in cancer immunotherapy [1,2,3,4]. The effectiveness of immunotherapy used in monotherapy, compared to chemotherapy, has been proven in first- and/or second-line treatment in patients with various types of cancer (melanoma, non-small-cell lung cancer, renal cell carcinoma, head and neck region cancer, urothelial carcinomas, colorectal cancer, esophageal cancer, and lymphoma) [1,2,3,4]. This situation occurs especially in clinically selected NSCLC patients without actionable driver mutations (*EGFR*, *ALK*, *ROS1*, *BRAF*, etc.) detected in tumour cells. In patients with non-small-cell lung cancer, pembrolizumab (anti-PD-1 antibody) and atezolizumab (anti-PD-L1 antibody) used as first-line therapy may only be appropriate for patients with PD-L1 expression on ≥50% of tumour cells (in the US, pembrolizumab can also be used in patients with a high tumour mutational burden and PD-L1 expression on ≥1% of tumour cells) [2,5,6,7]. Many clinical trials have shown the effectiveness of anti-PD-1/or anti-PD-L1 immunotherapy, compared to docetaxel, in second-line treatment of NSCLC patients regardless of the PD-L1 expression. In clinical practice, atezolizumab, nivolumab, and pembrolizumab are used in this indication [1,2]. Unfortunately, the benefits of treatment with pembrolizumab or atezolizumab in monotherapy do not accrue to all PD-L1-positive patients. Indeed, the PD-L1 expression on cancer cells is the only biomarker validated in prospective immunotherapy-based clinical trials; however, it is not an ideal one [8,9,10]. Preclinical experiments have found synergistic effects of various treatment strategies that, when used in combination with immunotherapy, can enhance its effectiveness. The aim of combination therapy is to create a favourable environment within the cancerous tumour and maximize the potential of the immune system to eliminate cancer cells [11]. Figure 1 shows that many anticancer therapies are currently a combination of two methods of treatment employed to maximize the effectiveness of such a therapeutic approach.

The idea of using two different immunotherapies in cancer patients is based on the attempt to stimulate or inhibit different immune cells at different levels of their activity (e.g., in the lymph node and in the tumour) [11,12,13,14,15]. The most commonly used combination immunotherapy involves antibodies that target molecules capable of stimulation of the activity of lymphocytes and other immune cells and molecules that are able to inhibit this activity. Another combination immunotherapy method is the use of immune checkpoint inhibitors in combination with agents that modify the tumour microenvironment in a non-specific manner (e.g., pro-inflammatory cytokines, immunosuppressive cytokine inhibitors, and indoleamine 2,3-dioxygenase and adenosine inhibitors) [11,12,13,14,15].

## 2. Possibilities of Combining Different Immune Checkpoint Molecules

### 2.1. Strategies to Combine Two Different Antagonistic Antibodies against Inhibitory Immune Checkpoints

The use of various ICIs has found the widest application in clinical practice in cancer patients without the presence of actionable mutations and based on tumour histology as well as specific clinical characteristic of patients. A summary of the most important clinical trial results from phase 2/3 using combination immunotherapies and their clinical efficacy is presented in Table 1 [16,17,18].

Dual blockade of PD-1 and CTLA-4 with nivolumab and ipilimumab has been used to treat melanoma, renal cell carcinoma, and non-small-cell lung cancer in clinical trials [19,20,21]. In the CheckMate 227 study, patients with advanced NSCLC were treated with a combination of nivolumab and chemotherapy or nivolumab and ipilimumab or with chemotherapy [22,23]. Two predictive markers for immunotherapy were used: PD-L1 expression on tumour cells and the number of somatic mutations in tumour cells (tumour mutation burden, TMB) [22,23]. It was found that, in patients with high TMB (more than 10 mutations per million base pairs) even with no PD-L1 expression on tumour cells, the use of the nivolumab and ipilimumab combination prolonged progression-free survival, compared to other treatments [22,23]. During further follow-up, prolongation of patient survival was observed in patients with PD-L1 expression on ≥1% of tumour cells using the combination of these two immunotherapies. In view of these results, the combination of nivolumab and ipilimumab for first-line therapy in NSCLC patients with high TMB was not registered and replaced by the registration of the combination of these two drugs in NSCLC patients with any PD-L1 expression on tumour cells [22,23]. In addition, the first-line combination therapy involving ipilimumab, nivolumab, and two lines of chemotherapy was registered for patients with advanced NSCLC based on the results of the CheckMate 9LA study [22,23,24]. However, it should be also mentioned about the results of ipilimumab with pembrolizumab combination based on Keynote-598 study. This combination does not improve clinical efficacy in metastatic NSCLC patients with PD-L1 TPS ≥ 50% and no targetable *EGFR* or *ALK* aberrations. Moreover, this therapy was associated with greater toxicity than pembrolizumab monotherapy. However, in patients with high PD-L1 expression on tumor cells, immunotherapy alone appears to be a better therapeutic option [25].

There are ongoing clinical trials investigating the possibility of administration of durvalumab and tremelimumab (anti-CTLA-4 antibody) combinations in NSCLC patients [26]. It should also be mentioned at this point that the PD-L1 expression on cancer cells is the only predictive factor validated in prospective clinical trials for immunotherapy in advanced NSCLC patients [2,27]. However, based on the clinical trial results, it is also known as not an ideal predictive marker. Not all patients with high PD-L1 expression can benefit from immunotherapy, but a clinical response may also be observed in patients without PD-L1 expression [2,27,28]. The anti-tumour immune response is an extremely complex multi-stage process depending on many factors. Moreover, it has been indicated that tumours have three immunoprofiles based on the activation of the immune system: (1) “hot” tumours, which are strongly infiltrated by T lymphocytes and with many inflammatory signals; (2) “cold” tumours, which are scanted of any immune cells infiltration nor inflammatory signs; (3) tumours with immune exclusion, where immune cells are at the periphery or within the stromal tissue [29,30]. The ”hot” tumours are associated with denser PD-1-positive T lymphocyte infiltration, with pre-existing primed immune response, and are more likely to respond to the anti-PD-1 or anti-PD-L1 blockade used as monotherapy [29,30]. Other factors such as diet, body mass index, microbiome, lipid metabolism, and leptin activity have been shown to exert an influence on immunotherapy effectiveness [27]. What about combination therapy? In the IMpower 150 study, a significantly longer median progression-free survival was observed upon administration of combination therapy (atezolizumab, bevacizumab, chemotherapy) in patients with no PD-L1 expression but with low expression of T-effector activation genes than in patients receiving only bevacizumab with platinum doublets [31]. It may be speculated that the combination therapy triggered the release of tumour antigens, which contributed to the activation of the immune system. In addition, the PD-L1 molecule blockade may have inhibited the impact of the tumour on the immune system, stimulating it to fight effectively [29,30]. Therefore, the intensity of lymphocyte infiltration of tumour tissue, immunological analysis, or estimation of the gene expression profile in cancer tissue could be considered as a reliable biomarker in the prospective qualification for immunotherapy in different strategies.

### 2.2. Side Effects of Therapy Based on Combining Two Different Antagonistic Antibodies against Inhibitory Immune Checkpoints

Combination therapy with two different immunotherapy modalities is usually fairly well tolerated. Clinical trials did not identify a significant increase in the incidence of adverse events (AEs) in groups of patients treated with combination immunotherapy compared to monotherapy [16,17,18]. On the other hand, combination therapy with two ICIs causes a different type of side effects compared to chemotherapy. Patients receiving immunotherapy most often experience side effects related to hyperactivity of the immune system (endocrinopathies, pneumonitis, hepatotoxicity, skin reaction, and others), while patients receiving chemotherapy develop bone marrow suppression (anaemia, infections, thrombocytopenia, and febrile neutropenia) [16,17,18].

In the CheckMate 227 clinical trial, the frequency of grade 3 or 4 AEs was similar in the group that received nivolumab plus ipilimumab and in the chemotherapy group (32.8% vs. 36.0%) [17]. Serious treatment-related adverse events and AEs leading to discontinuation were more common in patients treated with nivolumab plus ipilimumab than with chemotherapy (24.5% vs. 13.9% and 18.1% vs. 9.1%). The most common treatment-related adverse events (TRAEs) of any grade related to the immune system in the group that received nivolumab plus ipilimumab were skin reactions (34.0% of the patients) and endocrinopathies (23.8%). Treatment-related deaths occurred in eight patients who received nivolumab plus ipilimumab and in six patients who received chemotherapy [17]. In patients with PD-L1 expression on ≥1% of tumour cells treated with nivolumab monotherapy, grade 3 or 4 TRAEs occurred in 19.4% of the patients, and TRAEs resulted in discontinuation of the therapy in 12.3% of the patients. Two treatment-related deaths occurred in the nivolumab monotherapy group. In patients without expression of PD-L1 treated with nivolumab plus chemotherapy, serious TRAEs occurred with a frequency of 19.2%. Four deaths were reported in this group [17].

In the CheckMate 9LA clinical trial, serious TRAEs were reported in 30% of patients receiving combination therapy and in 18% of patients treated with chemotherapy [16]. Seven (2%) treatment-related deaths were observed in the former group. The following causes of death were found: acute kidney failure, colitis with diarrhoea, hepatotoxicity, hepatitis, pneumonitis, sepsis with acute renal insufficiency, and thrombocytopenia. Six (2%) deaths due to anaemia, febrile neutropenia, pancytopenia, pulmonary sepsis, respiratory failure, and sepsis occurred in the control group [16]. The most common grade 3–4 TRAEs were neutropenia (7% of patients treated with combined therapy vs. 9% of patients receiving chemotherapy), anaemia (6% vs. 14%), diarrhoea (4% vs. 1%), and febrile neutropenia (4% vs. 3%). These TRAEs were associated with the use of chemotherapy rather than immunotherapy [16].

In the CITYSCAPE clinical trial, grade ≥3 TRAEs occurred in 19.1% of patients treated with atezolizumab monotherapy and in 14.9% of patients receiving atezolizumab in combination with tiragolumab [18]. AEs leading to treatment withdrawal occurred in 10.3% of patients from the former group and 7.5% of patients from the latter group [18].

In conclusion, the development of certain equilibrium between the effectiveness of combination therapy and its side effects should be considered. In most cases, when the side effects of combined therapy are detected at an early stage and are not very severe, it is possible to protect the patient properly against their consequences. It can be speculated that this should bring clinicians closer to the use of combination therapy in the clinic.

### 2.3. Molecular and Immunological Synergy of Antagonistic Antibodies against Different Inhibitory Immune Checkpoints

**Figure 2:**
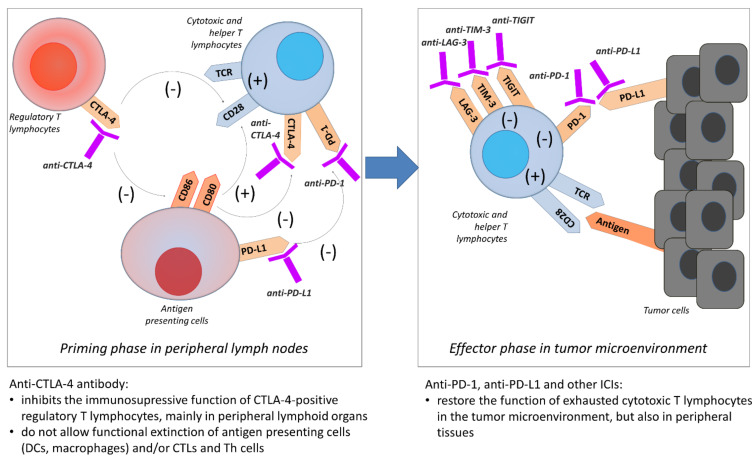
Mechanism of the activity of most important immunological checkpoints.

The effectiveness of combination therapy with nivolumab and ipilimumab is explained by the presence of interactions of these antibodies on different immunological checkpoint molecules [13,32,33,34,35]. Nivolumab inhibiting the PD-1 receptor causes activation of T lymphocytes in the tumour, lymph nodes, and peripheral tissues. This is related to the fact that the PD-L1 molecule is present on tumour cells (in primary tumours and metastases), on antigen-presenting cells infiltrating the tumour and occurring in lymph nodes (also normal, which limits the development of uncontrolled inflammatory reaction), and on most normal cells (limitation of autoimmune reaction) [13,15,32,34]. The function of the CTLA-4 molecule found on the surface of T lymphocytes is quite different [21,36]. Its stimulation plays a role during the induction of the immune response at the stage of antigen presentation. The CTLA-4 instead of CD28 molecule (the main costimulatory molecule) binds with CD80 and CD86 molecules on APC, which inhibits proliferation and activation of T helper and cytotoxic lymphocytes [15,21,36,37]. Furthermore, this interaction leads to the exfoliation of CD80 and CD86 molecules from the surface of antigen-presenting cells, causing their non-functionality. High expression of CTLA-4 on T lymphocytes also induces the intracellular FoxP3 (forkhead box P3) protein, resulting in the transformation of these cells into T regulatory lymphocytes. These reactions occur to the greatest extent in lymph nodes [15,21,36,37]. According to these considerations, the synergistic effect of nivolumab and ipilimumab consists of enhancement of the activation of T helper and cytotoxic lymphocytes by blocking one of the most potent signals inhibiting these cells (PD-1 and PD-L1 interaction) and restoring the most important, besides antigen presentation, costimulatory signal (CD28-CD80 and CD86 connections) [15,21,36,37]. Moreover, the use of ipilimumab further reduces the immunosuppressive effect of other cells of the immune system [15,19,21]. The schematic mechanism of the activity of the most important immunological checkpoints is illustrated in Figure 2.

In laboratory studies, this interaction between these two ICIs is strongly expressed. In the peripheral blood of patients treated with the combination therapy, compared to nivolumab or ipilimumab monotherapy, the percentage of T cytotoxic lymphocytes is significantly increased [38,39,40]. High levels of the proinflammatory cytokines sIL-2Rα, IL-1α, and chemokines (e.g., CXCL10) are noted in the plasma of patients undergoing combined immunotherapy, which cannot be achieved with nivolumab or ipilimumab alone. Patients with a response to combination therapy show an increase, relative to the level before the therapy, in the percentage of memory T cytotoxic lymphocytes with an EOMES+ (eomesodermin), CD69+, CD45RO+ phenotype. In addition, low expression of other negative immune checkpoints, most notably TIGIT and lymphocyte-activation gene 3 (LAG3), is observed on lymphocytes in patients responding to such treatment [38,39,40,41]. This phenomenon is not observed in patients responding to nivolumab monotherapy. The analysis of the expression of genes responsible for the immune response profile in peripheral blood leukocytes was carried out as well. Patients undergoing combination therapy express genes for granzymes A/B, Ki-67, IL-8, and HLA-DR (Human Leukocyte Antigen—DR isotype), which indicates cytolytic and proliferative activity of T cytotoxic lymphocytes and their ability to infiltrate tumour tissue. Patients receiving anti-PD-1 antibodies have increased expression of genes determining the cytolytic activity of lymphocytes (genes for granzymes A/B, KLRF1, FCRL3) [37,38,39,40]. In turn, increased expression of genes related to the capability of T lymphocytes of proliferation and production of specific cytokines (genes for Ki-67 and ICOS) is detected in patients receiving ipilimumab [38,39,40,41].

In a mouse model, tumour-infiltrating T cytotoxic lymphocytes have been divided according to their immunophenotype into 4 groups: (1) T lymphocytes with a functionally depleted cell phenotype (PD-1^high^, LAG3^++^, TIM3^++^), (2) terminally differentiated T lymphocytes with an activated phenotype (PD-1^+^, LAG3^int^, TIM3^int^), (3) T lymphocytes at an early stage of differentiation (Tbet^int^, CD86^+^, PD-1^+/−^, Bcl2^+^), and (4) apoptosis-resistant migratory T lymphocytes (PD-1^−^, CD62L^+^, Bcl2^++^) [13,38]. The use of combination immunotherapy, compared to nivolumab or ipilimumab monotherapy, significantly increases the percentage of differentiated and activated lymphocytes and significantly decreases the percentage of functionally depleted lymphocytes [13,38]. However, the type of therapy has no effect on the percentages of other T cytotoxic lymphocyte subpopulations in the peripheral blood. Among the T helper lymphocytes, subpopulations differing in the immunophenotype have also been distinguished: Th1 lymphocytes with an effector phenotype (PD-1^+^, GATA3^+^, CD44^+^, CXCR3^++^), T lymphocytes with a helper phenotype without chemokine receptors (CD44^+^, GATA3^+^, CD44^+^, CXCR3^−^), and actively migrating T lymphocytes that resist apoptosis (PD-1^−^, CD62L^+^, Bcl2^++^) [13,38,39,40,41,42]. Combination therapy, compared to nivolumab or ipilimumab monotherapy, results in significantly increased infiltration of Th1 effector lymphocytes. T regulatory lymphocytes can be divided into three groups according to their immunophenotype: (1) Treg lymphocytes with a pro-tumour phenotype (CTLA-4^++^, FoxP3^+^, CD25^+^), (2) Treg lymphocytes with an incomplete differentiation phenotype (CTLA-4^+^, FoxP3^++^, CD25^++^), and (3) undifferentiated and depleted Treg lymphocytes (CTLA-4^−^, FoxP3^+/−^, CD25^++^). A lower degree of infiltration of Treg lymphocytes with a pro-tumour immunophenotype was detected in mice treated with ipilimumab or combination therapy compared to nivolumab-treated or untreated mice. At the same time, it was shown that the percentage of Th1 effector lymphocytes correlated negatively and the percentage of pro-tumour Treg lymphocytes correlated positively with tumour size [41].

Based on these theoretical considerations and laboratory study results, quite new concepts of clinical trials combining antibodies that interact with different immune checkpoints have been developed. There are ongoing clinical trials in patients with advanced NSCLC, in which classical anti-PD-1 or anti-PD-L1 antibodies are attempted to be combined with antibodies against ICOS (inducible T-cell costimulator), LAG-3, TIM-3 (T-cell immunoglobulin domain and mucin domain 3), or TIGIT [43,44,45,46,47]. Research on new anti-LAG3 and anti-TIGIT antibodies is of particular importance. As noted above, patients without response to nivolumab and ipilimumab combination therapy had a significantly higher percentage of T lymphocytes with expression of these molecules. This suggests that their presence may have a leading role in inhibiting T lymphocyte activation and in inducing resistance to existing immunotherapies [43,44,45,48]. Therefore, there are indications for replacement of the anti-CTLA-4 therapy in combination therapy using anti-PD-1 or anti-PD-L1 antibodies with anti-LAG3 or anti-TIGIT antibodies [49]. A phase I trial in which tiragolumab (anti-TIGIT antibody) was used along with atezolizumab in patients with advanced NSCLC provided particularly interesting results [50,51,52]. Response to this type of therapy was achieved in 46% of patients, and disease stabilization occurred in 85% of patients. These encouraging results contributed to the initiation of phase II trial—CITYSCAPE and phase III trial—SKYSCRAPER-01, which used combination therapy with atezolizumab and tiragolumab compared to therapy with atezolizumab alone in advanced NSCLC patients with PD-L1 expression on tumour cells [18,53]. The CITYSCAPE trial demonstrated response in 31.3% of patients treated with the combination therapy and in 16.2% of patients receiving atezolizumab alone. The median progression-free time in these two patient groups was 5.4 months and 3.6 months, respectively [18,53].

### 2.4. Strategies to Combine Different Antagonistic and Agonistic Antibodies against Immune Checkpoints

On the other hand, there are ongoing early clinical trials in which agonistic antibodies that bind to costimulatory molecules on lymphocytes have been combined with antagonistic antibodies directed against negative checkpoints (usually anti-PD-1, anti-PD-L1, or anti-CTLA-4) [45,54]. Activation of CD28, CD27, OX40, CD137 (4-1BB), or GITR (glucocorticoid-induced TNFR-related) molecules increases lymphocyte proliferation and positively stimulates the development of immune response [55,56,57,58]. However, the use of agonist antibodies that bind to these molecules often causes serious side effects. Nevertheless, promising results have been obtained in cancer patients using a combination of classical ICIs with antibodies stimulating CD27 and CD137 activity [59].

The CD27 activation is a potent costimulatory factor in the first stages of immune response when it promotes T cell survival and memory T cell formation [60,61,62]. The only ligand for CD27 is the CD70 molecule found on APCs and on activated T lymphocytes. However, the interaction between CD27 and CD70 changes over the course of immune responses [63,64]. Chronic stimulation of CD27 by CD70 in chronic inflammation suppresses the immune response and, in the case of tumour cells expressing CD70, leads to differentiation of T lymphocytes into Treg cells [65,66]. A phase I/II clinical trial consisted in the use of varlilumab, i.e., an agonistic antibody that binds to CD27, in combination with nivolumab in patients with solid tumours [67,68]. Response to the treatment was achieved in 49% of patients, although most of them did not have PD-L1 expression on tumour cells. It turned out that, after 4–6 weeks of therapy, 76% of patients acquired PD-L1 expression on antigen-presenting cells. On the one hand, T lymphocytes were stimulated by activation of the CD27 molecule and, on the other hand, a purpose for nivolumab therapy (PD-L1 expression) emerged [67,68].

## 3. Use of Non-Specific Immune System Stimulation and Tumour Microenvironment Modification in Immune Combination Therapies

Non-specific immunotherapy can also be associated with immune checkpoint inhibitors. Non-specific stimulation of the cytotoxic response against tumour cells can be achieved by administration of proinflammatory cytokines or by inhibition of the immunosuppressive cytokine function [69,70]. In the first case, clinical trials have been undertaken to assess combination therapy of cancer patients with anti-PD-1 and anti-PD-L1 antibodies in combination with modified cytokines IL-2 and IL-15 [69]. Pegylated IL-2 with attached polyethylene glycol chains (bempegaldesleukin) has a longer half-life in the body than recombinant IL-2 (aldesleukin) [71,72]. Bempegaldesleukin binds to heterodimeric IL-2Rβγ (CD122), which preferentially activates effector cytotoxic T lymphocytes and NK cells in the peripheral blood and tumour microenvironment. In contrast, pegylated IL-2 has a low affinity towards the receptor for IL-2 built of alpha, beta, and gamma subunits (IL-2Rαβγ, CD25), which is mainly found on Treg cells [71,72,73]. Due to these properties, bempegaldesleukin does not activate T lymphocytes and NK cells immediately after infusion and only transiently activates Treg cells, resulting in a higher safety profile compared to that of aldesleukin [71,72,73].

Clinical studies on the use of recombinant IL-15 have also been undertaken. However, this molecule was quickly replaced by an IL-15 superagonist (ALT-803), which consists of a modified IL-15 molecule with an introduced N72D mutation, a modified receptor for IL-15 (IL-15R), and an Fc fragment of IgG1 class antibody linking everything [74,75,76,77]. The IL-15 molecule is supposed to bind to IL-2Rβγ in order to stimulate cytotoxic T lymphocytes and NK cells. The modified IL-15R ensures specific binding of ALT-803 to IL-2Rβγ, rather than to IL-2Rαβγ, which is found on Treg cells, while the Fc fragment of the antibody prolongs the half-life of the complex and attracts NK cells [74,75,76,77,78].

Bempegaldesleukin and ALT-803 have been used in combination with nivolumab and atezolizumab in patients with various types of cancer (including hematologic) in phase I clinical trials with promising results and satisfactory safety [79,80]. In turn, therapies in which anti-PD-1, anti-PD-L1, or anti-CTLA-4 antibodies were combined with therapies aimed at reducing the activity of immunosuppressive cytokines such as TGF-β (tumour growth factor beta), M-CSF (macrophage-colony stimulating factor), and IL-10 seem to be less effective [81,82]. Addition of drugs blocking IL-10 or TGF-β function to classical immunotherapy increased the risk of adverse effects in the form of autoimmune reactions [83,84,85,86]. Nevertheless, clinical trials are underway to investigate the effectiveness of M7824—a fusion protein consisting of a human IgG1 monoclonal antibody against PD-L1 fused to the extracellular domain of the receptor for TGF-β, which captures TGF-β in the tumour environment [86]. This drug may have great potential in the treatment of cancer patients in combination with other immunotherapies (e.g., anti-CTLA-4), in first-line monotherapy, and in combination with chemotherapy. It has selective effects in PD-L1 positive tumours and has fewer side effects than other anti-TGF-β agents [86].

The tumour microenvironment has a very adverse effect on the immune system functioning therein [83]. An unfavourable tumour microenvironment results in exclusion of immune response outside the tumour. Two substances play a special role here. One of them is adenosine [87,88]. Adenosine and ATP are present at exceptionally low concentrations in extracellular fluids. However, inflammation, ischemia, or the cancer process can lead to the release of ATP through transport channels in cell membranes, active exocytosis, and directly from damaged cells [89,90,91]. Extracellular ATP acts as a danger-associated molecular pattern (DAMP) to promote the immune response. However, during inflammation, extracellular ATP is progressively dephosphorylated by ectonucleotidases (mainly CD39 and CD73), resulting in the formation of adenosine. Adenosine binds to its receptors A1, A2a, A2b, and A3. Stimulation of the A2a receptor inhibits the cytotoxic T cell activity and promotes the Treg cell activity by increasing FoxP3 expression. Under the influence of this stimulation, the expression of immune checkpoints including PD-1, CTLA-4, and LAG-3 increases on effector lymphocytes. Therefore, it is not surprising that molecules that block adenosine binding to the A2a receptor and molecules that inhibit the activity of the CD39 and CD73 enzymes have been developed and used in combination with anti-PD-1 or anti-CTLA-4 antibodies in early phase clinical trials in cancer patients [89,90,91].

Another substance that causes elimination of tumour cells from the tumour area is indoleamine 2,3-dioxygenase (IDO) [92,93,94,95]. This enzyme metabolizes tryptophan to kynurenine. The production of IDO by tumour cells reduces tryptophan levels in the tumour. Tryptophan, i.e., an exogenous amino acid, is essential for normal lymphocyte function. Its absence in the tumour environment prevents T lymphocytes from entering the tumour [92,93,94]. Studies on the possibility of combining IDO inhibitors (e.g., epacadostat) with classical ICIs in NSCLC and melanoma patients have been conducted for several years. However, phase III trials failed to demonstrate the effectiveness of such therapy, which resulted in the lack of registration of epacadostat in combination with pembrolizumab for the treatment of melanoma and NSCLC patients [92,93].

## 4. Conclusions

Standard anti-cancer therapies, such as radiotherapy or chemotherapy, destabilize tumour cell function, contribute to the release of tumour antigens and the formation of neoantigens, and affect the production of cytokines, chemokines, and other substances that stimulate immune cell activity. As a result, tumours with low immunogenicity (“cold”) could be transformed into tumours with high immunogenicity (“hot,” “inflammatory”), abundant with infiltrates of activated specific lymphocytes [29,30]. This breaks down the mechanism by which tumour cells escape from immune surveillance. The addition of immunotherapy targeting immune checkpoints to chemotherapy or chemoradiotherapy further enhances the antitumor effects of cytotoxic T lymphocytes.

On the other hand, combining two different immunotherapy methods in cancer patients may be as effective as chemoimmunotherapy or chemoradiotherapy in cancer therapy. The combination of two immunotherapy methods is based on the idea of stimulating or inhibiting different immune cells at different levels of their activity with two different immune point activators or inhibitors, or using conventional ICIs in combination with non-specific immunostimulatory agents or agents that modify the tumour microenvironment. However, patients should be very well suited to this type of treatment. At present, there are no conclusively proven predictors for combination therapies, but the selection of patients should be based on clinical factors, such as the performance status of the patients, the presence of comorbidities, and the availability to a multidisciplinary cancer centre, which is extremely important for the proper management of patients.

Currently, scientists have a wide range of possibilities to investigate and combine different therapeutic approaches. The treatment method based on the use of specific genetically modified CAR-T cells (chimeric antigen receptor) is developing dynamically. Attempts are underway to combine classical immunotherapy targeting immune checkpoints with treatment using modified oncolytic viruses. Already, the median survival of patients with advanced non-small-cell lung cancer has increased significantly. The development of modern personalized treatments, including immunotherapies, enables many patients to act in good functional status for 3 years and beyond. In the near future, it is expected that many patients will live with cancer just as patients with cardiovascular or infectious diseases (e.g., AIDS and hepatitis C) are currently living in near-complete comfort.

In conclusion, combination immunotherapies will be used in cancer patients, not only those with lung cancer. Therefore, is seems extremely important to understand the mechanisms of action of combined immunotherapy, firstly to understand how these therapies work in the patient’s body and, secondly, to be able to quickly recognize the side effects and properly secure the patients.

## Figures and Tables

**Table 1:** The Summary of the most important clinical trial results using combination immunotherapies. Abbreviations: ORR—overall response rate, PFS—progression free survival, HR—hazard ratio, CI—confidential interval, OS- overall survival, PD-L1—programmed death ligand 1, TC—tumor cells, ND—no data. (* PD-L1 expression examined by 22C3 monoclonal antibody; ** PD-L1 expression examined by SP263 monoclonal antibody) [16,17,18].

Clinical Trial Identifier	Phase	Predictive Factor	Stage of NSCLC	Drugs	Number of Patients	ORR (%)	Median PFS (months)	PFS (HR, 95% CI)	Median OS	OS (HR, 95% CI)
CheckMate 227 NCT02477826	3.	≥1% of PD-L1-positive TC (Part 1a)	IV	Nivolumab	396	27.5	4.2	0.82, 0.69–0.97 (nivolumab + ipilimumab vs. chemotherapy) 0.83, 0.71–0.97 (nivolumab + ipilimumab vs. nivolumab)	15.7	0.79, 0.65–0.96 (nivolumab + ipilimumab vs. chemotherapy) 0.90, 0.76–1.07 (nivolumab + ipilimumab vs. nivolumab)
				Nivolumab + ipilimumab	396	35.9	5.1		17.1	
				Chemotherapy	397	30	5.6		14.9	
CheckMate 227 NCT02477826	3.	<1% of PD-L1-positive TC (Part 1b)	IV	Nivolumab + chemotherapy	177	37.9	5.6	0.75, 0.59–0.96 (nivolumab + ipilimumab vs. chemotherapy) 0.98, 0.77–1.24 (nivolumab + ipilimumab vs. nivolumab + chemotherapy) 0.73, 0.56–0.95 (nivolumab + chemotherapy vs. chemotherapy)	15.2	0.62, 0.48–0.78 (nivolumab + ipilimumab vs. chemotherapy) 0.77, 0.60–0.98 (nivolumab + ipilimumab vs. nivolumab + chemotherapy) 0.78, 0.60–1.02 (nivolumab + chemotherapy vs. chemotherapy)
				Nivolumab + ipilimumab	187	27.2	5.1		17.2	
				Chemotherapy	186	33.1	4.7		12.2	
CheckMate 227 NCT02477826	3.	All patients	IV	Nivolumab + ipilimumab	583	33.1	5.1	0.79, 0.69–0.91	17.1	0.73, 0.64–0.84
				Chemotherapy	583	27.7	5.5		13.9	
CheckMate 9LA NCT03215706	3.	All patients	IV	Nivolumab + ipilimumab + 2 cycles of chemotherapy	361	38.2	6.8	0.70, 0.57–0.86	15.6	0.66, 0.55–0.80
				Chemotherapy	358	24.9	5.0		10.9	
CITYSCAPER (NCT03563716)	2.	≥1% of PD-L1-positive TC	IIIB or IV	Chemotherapy	68	21% * 23% **	3.88 * 4.11 **	0.58, 0.39–0.88 * 0.56, 0.34–0.92 **	ND	ND
				Atezolizumab + tiragolumab	67	37% * 42% **	5.55 * 10.18 **		ND	

## Data Availability

Data sharing not applicable. No new data were created or analyzed in this study. Data sharing is not applicable to this article.
